# Resignation of Professor Moorhead

**Published:** 1847-05

**Authors:** 


### RESIGNATION OF PROFESSOR MOORHEAD.

Dr. Moorhead has resigned the Chair of Theory and Practice of Medicine, which he has held for many years in the Ohio Medical College.

